# Copper(II) and Cobalt(II) Complexes Based on Abietate Ligands from *Pinus* Resin: Synthesis, Characterization and Their Antibacterial and Antiviral Activity against SARS-CoV-2

**DOI:** 10.3390/nano13071202

**Published:** 2023-03-28

**Authors:** Jamille de S. Correa, Julia de O. Primo, Nayara Balaba, Christoph Pratsch, Stephan Werner, Henrique E. Toma, Fauze J. Anaissi, Ruddy Wattiez, Cristina M. Zanette, Rob C. A. Onderwater, Carla Bittencourt

**Affiliations:** 1 Departament of Chemistry, Universidade Estadual do Centro-Oeste, UNICENTRO, Alameda Elio Antonio Dalla Vecchia, 838, Guarapuava 85040-167, PR, Brazil; 2 Department X-ray Microscopy, Helmholtz-Zentrum Berlin für Materialien und Energie GmbH, Albert-Einstein-Str. 15, 12489 Berlin, Germany; 3 Institute of Chemistry, University of São Paulo, São Paulo 05508-000, SP, Brazil; 4 Department of Chemistry, University of Mons, Place du Parc 23, 7000 Mons, Belgium; ruddy.wattiez@umons.ac.be; 5 Department of Food Engineering, Universidade Estadual do Centro-Oeste, UNICENTRO, Alameda Elio Antonio Dalla Vecchia, 838, Guarapuava 85040-167, PR, Brazil; 6 Materia Nova ASBL, 7000 Mons, Belgium

**Keywords:** antimicrobial pigments, antiviral surfaces, SARS-CoV-2, abietic acid, natural resin

## Abstract

Co-abietate and Cu-abietate complexes were obtained by a low-cost and eco-friendly route. The synthesis process used *Pinus elliottii* resin and an aqueous solution of CuSO_4_/CoSO_4_ at a mild temperature (80 °C) without organic solvents. The obtained complexes are functional pigments for commercial architectural paints with antipathogenic activity. The pigments were characterized by Fourier-transform infrared spectroscopy (FTIR), mass spectrometry (MS), thermogravimetry (TG), near-edge X-ray absorption fine structure (NEXAFS), X-ray photoelectron spectroscopy (XPS), scanning electron microscopy (SEM), and colorimetric analysis. In addition, the antibacterial efficiency was evaluated using the minimum inhibitory concentration (MIC) test, and the antiviral tests followed an adaptation of the ISO 21702:2019 guideline. Finally, virus inactivation was measured using the RT-PCR protocol using 10% (*w*/*w*) of abietate complex in commercial white paint. The Co-abietate and Cu-abietate showed inactivation of >4 log against SARS-CoV-2 and a MIC value of 4.50 µg·mL^−1^ against both bacteria *Staphylococcus aureus* (*S. aureus*) and *Escherichia coli* (*E. coli*). The results suggest that the obtained Co-abietate and Cu-abietate complexes could be applied as pigments in architectural paints for healthcare centers, homes, and public places.

## 1. Introduction

The SARS-CoV-2 virus is responsible for the coronavirus disease 2019 (named COVID-19 by the WHO on 11 February 2020) pandemic. Coronaviruses (CoVs) are enveloped positive single−strand RNA viruses. CoVs are classified into four genera, α-, β-, γ- and δ-coronaviruses. β-CoV is responsible for Severe Acute Respiratory Syndrome (SARS) and SARS-CoV-2 (also known as 2019-nCoV) [1]. The SARS-CoV-2 genome encodes for a spike−like glycoprotein (S) outside the viral particle, where it can bind to a cellular receptor and mediate membrane fusion and virus entry [2]. The initial COVID-19 outbreak was reported in December 2019 in Wuhan, China, and expanded globally, infecting almost 35 million people and causing more than a million deaths [1], devastating the world’s economy [3]. The first vaccines reduced the number of hospitalizations, deaths, and infection incidence. However, disinfection methods play a significant role in controlling local transmissions [4]. A typical transmission route for SARS-CoV-2 is through viral-contaminated surfaces such as walls, door knobs, packaging, and handrails [5,6] because this virus can persist on various surfaces from hours to days [7]. Therefore, antiviral and antibacterial surfaces and coatings are being explored for application in multiple settings, such as healthcare centers, long−term care facilities, public transport, and schools [8].

Since ancient times, copper has been recognized for its biocidal properties to heal infections [9]. There were reports of the ancient Roman and Egyptian civilizations using copper-containing compounds as antimicrobial materials [10]. Copper and cobalt are antimicrobial metals widely used to date [8,11,12]. In addition, cobalt and copper have been reported as promising metals against COVID-19 infection due to their potent antiviral and antibacterial properties [13,14]. As a result, studies report applications of Co and Cu in the development of new Schiff base compounds, new materials for coating and disinfecting surfaces, and specific copper nanoparticles that have been doped in face masks [15,16].

Tricyclic diterpenoids of the abietane series and their natural and synthetic derivatives exhibit a broad spectrum of biological activities, for example, antibacterial, antiviral, anticonvulsant, antimalaria, antiulcer, anti-leishmaniasis, antioxidant, and others. Abietane acids, such as abietic acid commonly called silvic acid, and levopimaric acid, are found in the natural resin produced by the *Pinus* tree [17]. Natural resins are used in making fungicides, insecticides, fragrances, paints and solvents, adhesives, rubber, biofuels, and biodegradable products because they are easily obtained, inexpensive, and represent a renewable source material [18]. Recently, some studies have shown the antiviral potential of diterpenoids from *Pinus* resin, including abietic acid, against SARS-CoV-2 [19].

The acid functional group of the abietic acid molecule can react with metals by coordinating with oxygen atoms, forming stable metal carboxylate complexes. The transition metal complexes have been reported as antibacterial and antiviral agents against SARS-CoV-2, suggesting they are potential inhibitors of the spike protein [20]. However, they can involve distinct mechanisms of action, considering the several molecular geometries of the complexes, their ability to undergo ligand exchange reactions, and accessible redox processes [21].

Here, we report on the synthesis of copper and cobalt complexes with carboxylate species derived from *Pinus elliottii* var. *elliottii* using a green synthetic route. The complexes were evaluated as pigments in commercial paint. The antibacterial efficiency was assessed using the minimum inhibitory concentration (MIC) test, and the antiviral tests were performed by an adaptation of the ISO 21702:2019 guideline. Virus inactivation was measured using an RT-PCR-based protocol using 10% (*w*/*w*) of abietate complex in commercial acrylic paint.

## 2. Materials and Methods

### 2.1. Reagents, Materials, and Synthesis Procedure

For the metal−abietate synthesis, the resin of Pinus elliottii var. elliottii is supplied in nature by Guarapuava, Parana State, Brazil producers. This resin was purified following the method reported in ref. [22].

All reagents are from Sigma Aldrich (São Paulo, SP, Brazil) (puriss. PA). First, the purified *Pinus* resin and sodium hydroxide (NaOH, PA) were dissolved in water and mixed in a molar proportion of 1:1, with mechanically stirring for 3 h, at 90 °C, until the formation of a hygroscopic salt (Na-abietate), as reported in [23]. Next, the Na-abietate was macerated in an agate mortar with a pestle. Then Na-abietate and cobalt sulfate (CoSO_4_, P.A.) was dissolved separately in water. These solutions were mixed dropwise in molar proportions of 3:1 (Na-abietate: Co^2+^), forming the Co-abietate complex instantaneously. Next, this complex was washed out with deionized water, filtered, and dried in an oven at 70 °C for 5 h. Finally, the powder was characterized and applied as an antibacterial pigment in a commercial white paint. This procedure was repeated for copper, using CuSO_4_, to form Cu-abietate [24].

### 2.2. Material Characterization

Fourier transform infrared spectroscopy (FTIR) analyses were performed on a Perkin Elmer Frontier spectrometer (Pontyclun, Mid Glamorgan, UK) in the 4000–650 cm^−1^ region. The samples were analyzed in the Eco-ATR attenuated total reflectance acquisition mode, equipped with a high-capacity ZnSe ATR crystal to analyze powders, solids, pastes, and liquids. Eight scans were performed with a spectral resolution of 2 cm^−1^.

The mass spectra (MS) of abietate complexes were obtained from a solution of dichloromethane (DCM) diluted in methanol injected in a Bruker Amazon Speed ETD equipment from Bruker Daltonics, Billerica, MA, USA, ion trap (MS-MS) with low resolution, in negative ion and ionization by electrospray mode. A drying gas flow of 4 L·min^−1^ was used at a temperature of 200 °C, nitrogen as a nebulizer gas under pressure of 7 psi, and a voltage of 4500 V. 

The NEXAFS spectra were recorded at the XM-beamline (U41-PGM1-XM) at BESSY II, Berlin. The TXM optical design combines a spectral resolution of E/ΔE = 1 × 10^4^ with a spatial resolution of 25 nm in a field of view of 15–20 µm. The design of the X-ray microscopy beamline U41-PGM1-XM allows analysis in the soft (0.25–1.5 keV) and tender X-ray photon energy regime (1–2.7 keV) [25,26]. 

The chemical composition was evaluated by X-ray photoelectron spectroscopy (XPS) (Versaprobe PHI 5000 from Physical Electronics, Chanhassen, MN, USA), equipped with a monochromatized X-ray source Al Kα). The XPS spectra were collected at a takeoff angle of 45° with the electron energy analyzer, and the spot size was 200 µm. A passage energy of 20 eV was used for the high-energy resolution spectra recorded on the Co 2p, Cu 2p, O 1s, and C 1s core level energy range. The spectra were analyzed using the CASA-XPS software (Teignmouth, Devon, UK). 

Scanning electron microscopy (SEM) images were performed using a Hitachi TM-3000 Field Emission Scanning Electron Microscope (Tokyo, Japan) operated at 15 kV. The spatial resolution was 5 nm.

The Co-abietate and Cu-abietate samples, in powder form, were evaluated by colorimetry. The coordinates were determined by a portable colorimeter (model NR60CP, 3nh (Shenzhen, China)) with a D65 light source. The CIE 1976 L*a*b* colorimetric method was used. In this method, L* is the color lightness (L = 0 for black; and L = 100 for white), a* is the green (−)/red (+) axis, and b* is the blue (−)/yellow (+) axis, as recommended by the Commission Internationale de I’Eclairage (CIE) [27].

Thermal decomposition was analyzed on a Perkin Elmer thermal analyzer, STA 6000, in Simultaneous Differential Scanning Calorimetry (STA/TG-DSC) mode (from Pontyclun, Mid Glamorgan, UK). A heating rate of 10 °C/min was used, in the temperature range of 30 to 1000 °C, with the support of alumina samples, in a nitrogen atmosphere with an average flow rate of 40 mL·min^−1^. 

### 2.3. Antibacterial Test

The broth microdilution method measured the minimum inhibitory concentration (MIC) according to the adapted methodology from the Clinical Manual and Laboratory Standards Institute (CLSI, 2006). Inoculums of *E. coli* (ATCC 25922) and *S. aureus* (ATCC^®^. 25923) were cultivated at 35 °C for 18 h and diluted to obtain a density of 10^5^ CFU mL^−1^. Next, these extracts were diluted in dimethyl sulfoxide (DMSO) to reach a concentration range from 3.20 to 24.5 µg·mL^−1^. A volume of 150 µL of Mueller Hinton broth containing the inoculum and 50 µL of different dilutions of pigments were added in each well. Microplates were incubated at 35 °C for 24 h. Bacterial growth was detected by adding 10 μL of sterile aqueous solution (20 mg·mL^−1^) of triphenyltetrazolium chloride (TTC, Inlab, Brazil) after incubation at 35 °C for 30 min. The minimum inhibitory concentration (MIC) is defined as the lowest concentration of the abietic complexes to inhibit bacterial growth, as indicated by a reduction in the red color of the TTC [28]. The experiments were performed in triplicate.

### 2.4. Antiviral Test

The antiviral tests were conducted using the RT-qPCR protocol of the national COVID-19 detection service in a procedure for viral inactivation detection. SARS-CoV-2 viruses were isolated into Hank’s balanced salt solution from nasopharyngeal swabs of confirmed COVID-19 patients and stored at −80 °C until application. The samples were residuals from the initial COVID-19 testing platform at UMONS (University of Mons, Mons, Belgium) and came to the testing platform from all over the Hainault region of Belgium. The exposure phase of the antiviral test followed the ISO 21702:2019 guideline, while an RT-qPCR−based technique was used to quantify the surviving intact viral particles. Polyvinyl chloride specimens were made in a size of 25 mm × 25 mm for the test. The specimens were coated with acrylic coatings (as negative control), and the coatings containing Co-abietate and Cu-abietate were synthesized. Weathered copper plates were used as positive controls. The samples were placed in individual wells of a sterile 6-well plate in triplicate. Before the tests, the samples were sterilized by ultraviolet according to the standard procedure (15 min per side). A liquid volume of 100 μL in a virus concentration corresponding to a Ct value in RT-qPCR of approximately 22 was added to each surface. A 20 mm × 20 mm polyethylene film cover was placed on top of the liquid. The samples were inoculated with 100% relative humidity at 37 °C. After the incubation, intact viral particles were recovered in 200 μL of a viral recovery solution containing 5 M guanidinium thiocyanate, 40 mM dithiothreitol, 20 µg/mL glycogen, 1% Triton X-100, buffered with 25 mM sodium citrate to pH 8 was used. Using the manufacturers’ extraction protocol, the viral RNA was extracted with AMPure XP magnetic beads (Beckman, MA, USA) SARS-CoV-2 viral suspensions were tested using the RT-PCR kit (Takyon One-Step Rox Probe 5x MasterMix dTTP, Eurogentec, Belgium). The Ct values, and the number of cycles necessary to spot the virus, were generated via the RT-PCR test as viral load indicators. The amplification reactions were performed using TaqMan RT-PCR on a StepOne Plus real-time PCR system (Applied Biosystems, Thermo Fisher, USA). The primers used were SARS E_Sarbeco-F1 (ACAGGTACGTTAATAGTTAATAGCGT) and SARS E_Sarbeco-R2 (ATATTGCAGCAGTACGCACACA) with SARS E_Sarbeco-P1 (FAM-ACACTAGCCATCCTTACTGCGCTTCGBBQ) as a fluorescent probe for the E gene using the Eurogentec (Belgium) Mastermix containing ROX as the internal reference. The PCR conditions were as follows: the initial denaturation step, 48 °C for 10 min for reverse transcription, followed by 95 °C for 3 min, and then 45 cycles of 95 °C for 15 s, 58 °C for 30 s. The antiviral activity was performed in triplicate, and the results were expressed logarithmically. The antiviral activity was assessed by one-way analysis of variance (ANOVA) followed by Tukey’s test at a 5% level of significance.

## 3. Results and Discussion

### 3.1. Vibrational Spectroscopy (FTIR)

**Figure 1:**
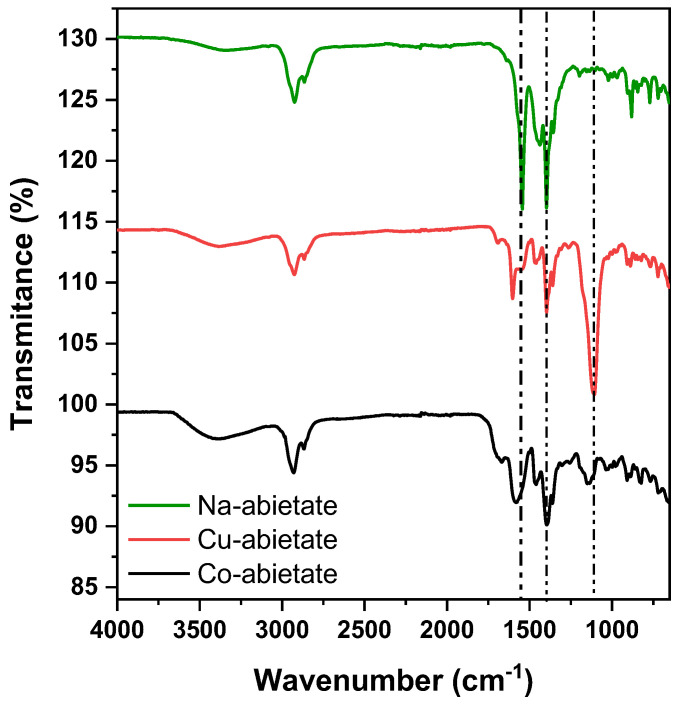
FTIR spectra of Na-abietate, the Co-abietate and Cu-abietate complexes.

The FTIR was used to analyze the abietate complex’s structure and the bonding mode. Figure 1 shows the FTIR spectra for the Co-abietate, Cu-abietate complexes, and the Na-abietate precursor. The characteristic bands obtained for FTIR of the metal-carboxylate interaction are represented by the strong asymmetric COO stretching vibration (υ_as_COO^−^) and the symmetric COO stretching vibration (υ_s_COO^−^) modes. The metal-carboxylate can coordinate in different ways, for instance, involving an ionic form, unidentate coordination, a bidentate chelating coordination, and a bidentate bridging coordination [29]. The wavenumber of the carboxylate bands varies according to the ligand and metallic core. The infrared spectra can access the binding mode of the carboxylate group by the difference in the wavenumbers of the symmetric and asymmetric modes, e.g., Δυ = υ_as_COO^−^ − υ_s_COO^−^. Analogously as shown by Deacon and Phillips [30], the ionic form (Na-abietate) can be used to determine the binding mode. This form is found in sodium or potassium salts. The carboxylate complex presents a Δυ(COO^−^) value different from the ionic form or carboxylate ion. The possible mode of coordination can be deduced by comparing the two forms. A general trend for band separation values, Δυ, can be outlined as uncoordinated acid > unidentate coordination > bidentate bridging > chelating coordination > free carboxylate ion (the ionic form) [31]. Table 1 shows the typical bands of carboxylate ligands for the Co-abietate and Cu-abietate complexes, the Na-abietate ionic form, including the Δυ(COO^−^) values. As can be seen in Table 1, the Δυ (COO^−^) of the Co-abietate complex exceeds the value for the ionic form (Na-abietate). However, the value is not exceptionally high, indicating that the complex is bidentate. The Cu-abietate complex showed a higher Δυ(COO^−^) value, consistent with a unidentate coordination mode. 

### 3.2. Mass Spectrometry (MS)

**Figure 2:**
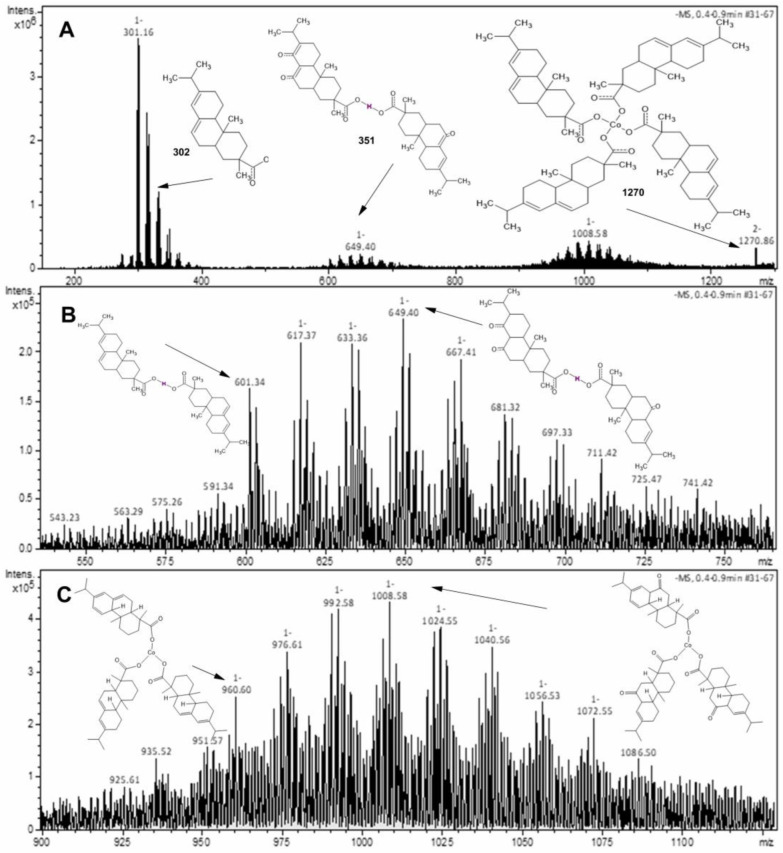
Mass spectra of the Co-abietate. (**A**) peaks corresponding to the theoretical molecular mass of deprotonated abietic acid, the dimeric form of the abietic acid with three additional oxygen atoms corresponding to oxidations of three C=C bonds to its keto form; the formation of Co-abietate with three abietate ligands with three additional oxygen atoms; and the formation of the Co-abietate complex with four abietate ligands at *m*/*z* 301, *m*/*z* 649, *m*/*z* − 1008, *m/z −* 1270, respectively. (**B**) The dimeric form of the abietic acid with three additional oxygen atoms from consecutive oxidations of C=C bonds, resulting in a difference of *m*/*z* 16 between at *m*/*z* − 617; *m/z −* 633; *m/z −* 649. (**C**) The peak of *m/z −* 1008 corresponds to Co-abietate formation encompassing three abietate ligands, with three C=C bonds from the ligand oxidated to the keto form.

Mass spectrometry is used to investigate the structure of the compounds. The mass spectra (MS) for Co and Cu-abietate were obtained from a solution of dichloromethane (DCM) diluted in methanol injected into an ion trap spectrometer (MS-MS) with low resolution in the negative and positive ion mode and electrospray ionization. Figure 2 shows the mass spectra of the Co-abietate sample and the respective structures corresponding to the *m*/*z* peaks in the negative ion mode. The prominent peak at *m*/*z* 301 corresponds to the theoretical molecular mass of deprotonated abietic acid [C_20_H_29_O_2_]. The peak at *m*/*z* 649 corresponds to forming a dimeric form of the abietic acid, but with three additional oxygen atoms corresponding to the oxidation of three C=C bonds to its keto form. Figure 2B shows these consecutive oxidations resulting in a difference of *m*/*z* 16 between them: *m*/*z* − 617; *m*/*z* − 633; *m*/*z* − 649. The peak of *m*/*z* − 1008 (Figure 2A) corresponds to the formation of Co-abietate with three abietate ligands [Co(C_20_H_29_O_2_)_3_]^−^ also with *m*/*z* equivalent to the complex with three additional oxygen atoms; these oxidations of the ligands are identified by *m*/*z* differences of 16 units between peaks (Figure 2C). The highest *m*/*z* peak at *m*/*z* − 1270 (Figure 2A) corresponds to the formation of the Co-abietate complex with four abietate ligands [Co(C_20_H_29_O_2_)_4_].

**Figure 3:**
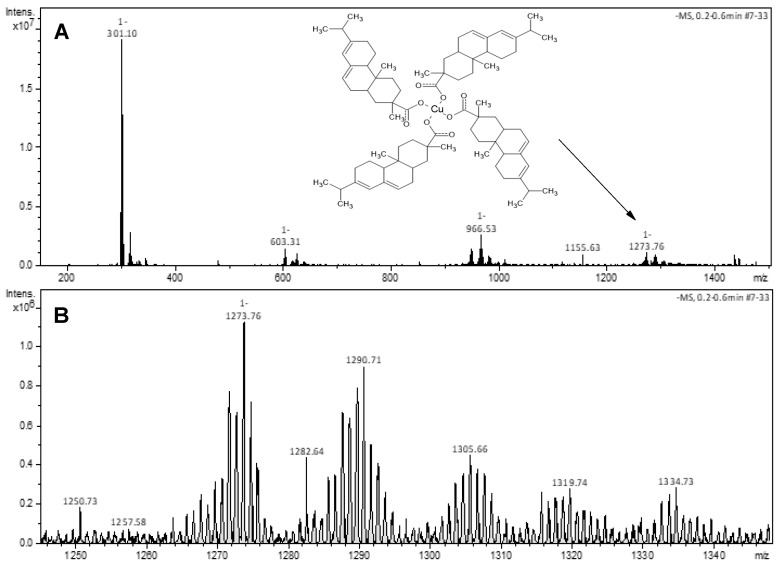
Mass spectra of the Cu-abietate. (**A**) The peaks corresponding to the molecular mass of deprotonated abietic acid (C_20_H_29_O_2_)^−^; to the dimeric form of abietic acid; the molecular mass of copper bound to three abietate ligands [Cu(C_20_H_29_O_2_)_3_]^−^; and to the Cu-abietate complex constituted by four ligands (C_20_H_29_O_2_)^−^, at corresponds to *m*/*z* − 301, *m*/*z* − 603, *m*/*z* − 966, and *m*/*z* − 1273, respectively. (**B**) Progressive oxidations of C=C bonds of the abietate ligand to the respective keto form, evidenced by the *m*/*z* difference of 16 units between the peaks.

The Cu-abietate sample shows *m*/*z* peaks with similar distribution to Co-abietate ones, suggesting a single metallic nucleus bond to four abietate ligands. This structure can be observed by the mass spectrum of the Cu-abietate compound (Figure 3A), in which four peaks are denoted: *m*/*z* − 301, *m*/*z* − 603, *m*/*z* − 966, and *m*/*z* − 1273, corresponding to the molecular mass of deprotonated abietic acid (C_20_H_29_O_2_)^−^; to the dimeric form of abietic acid; the molecular mass of copper bound to three abietate ligands [Cu(C_20_H_29_O_2_)_3_]^−^; and to the Cu-abietate complex constituted by four ligands (C_20_H_29_O_2_)^−^, respectively. Figure 3B shows the successive oxidations of C=C bonds, evidenced by the *m*/*z* difference of 16 units between the peaks. Compared to Co-abietate, the copper complex shows less unsaturation due to the different oxidative or reducing character of these transition metals.

### 3.3. NEXAFS

**Figure 4:**
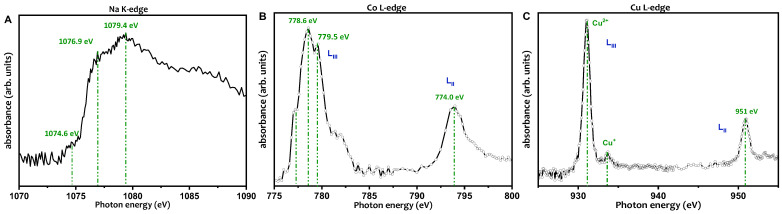
(**A**) Na K-edge NEXAFS of Na-abietate. (**B**) Co L-edge of Co-abietate. (**C**) Cu-L-edge of Cu-abietate.

The Na K-edge (Figure 4A) spectrum of the Na-abietate precursor presents three main features: the shoulder observed at 1076.9 eV is assigned to the 1s→3p transition [32], a peak at 1079.4 eV whose origin is not well established, and, a pre-edge structure observed in the spectrum at 1074.6 eV that corresponds to the transition 1s→3s [33]. This transition is parity forbidden in a free sodium ion. Therefore, it will not be observed in a NEXAFS spectrum of atomic sodium [32], indicating that the sodium atoms in the Na-abietate are in ionic form, as expected for this sample.

The X-ray absorption (NEXAFS) measurements at Co L-edge and Cu L-edge were performed to determine the electronic structure of the metals in Co-abietate and Cu-abietate samples. Figure 4B shows the absorption spectra of cobalt atoms in the Co-abietate sample. The NEXAFS spectrum results from the 2p→3d dipole transitions. The Co LIII absorption lines exhibit peaks centered at 777.1, 778.6, and 779.5 eV and the Co LII at 774.0 eV. These are characteristic of the Co^2+^ oxidation state [34,35,36,37].

The Cu 2p NEXAFS spectrum (Figure 4C) from the Cu-abietate shows the dipole transition of the Cu 2p3/2 (LIII) and 2p1/2 (LII) electrons into the empty d-states [38]. The intense absorption band at 931.1 eV is characteristic of the Cu^2+^ oxidation state. In contrast, the low-intensity peak at a photon energy of 933.7 eV is related to the presence of Cu^+^ in a smaller proportion [39]. The energy separation between LIII and LII features, which is determined by the spin coupling, is dependent on the oxidation state of Cu. It is 19.0 eV for Cu^2+^ and 21.0 eV for Cu^+^ [38]. However, for Cu-abietate in Figure 4C, the separation between LIII and LII is 20 eV and 18 eV for Cu^2+^ and Cu^+^, respectively. This difference occurred because the oxidation states mixture resulted in a peak (at 951 eV) displacement in the LII edge. It should be noted that Cu^2+^ ions were used in the Cu-abietate synthesis process, and the presence of Cu^+^ indicates a change in the electronic structure of the metal, i.e., the abietate ligand contributed to the reduction of a small portion of the copper ions used in the synthesis.

### 3.4. XPS

**Figure 5:**
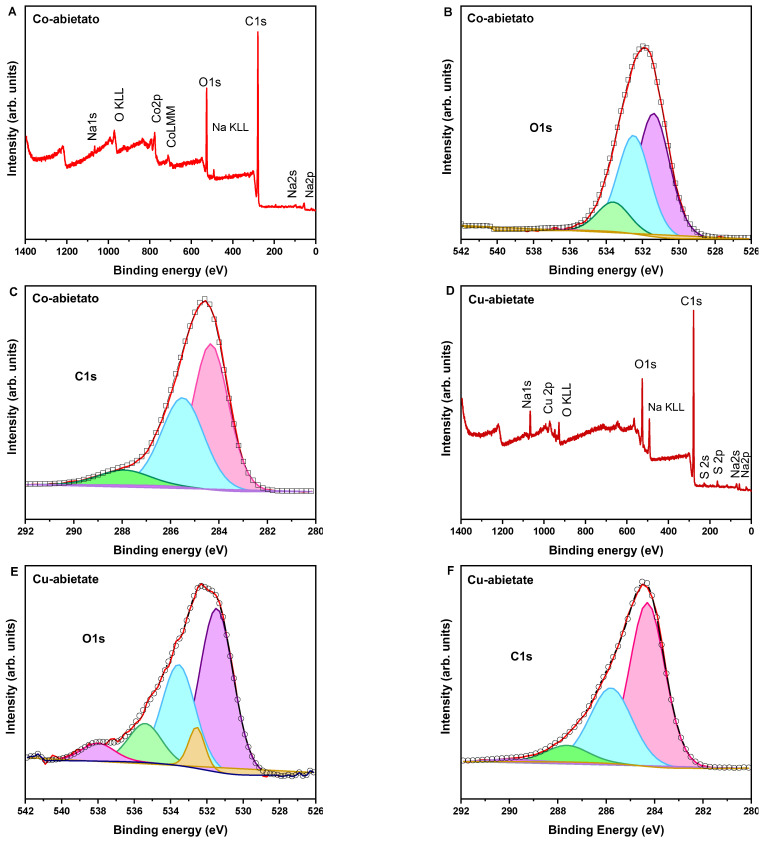
XPS spectra. (**A**) Survey spectrum of Co-abietate. (**B**) O1s spectrum of Co-abietate. (**C**) C1s spectrum of Co-abietate. (**D**) Survey spectrum of Cu-abietate. (**E**) O1s spectrum of Cu-abietate. (**F**) C1s spectrum of Cu-abietate.

The composition of Co-abietate and Cu-abietate was evaluated by X-ray photoelectron (XPS). The spectra were analyzed using the CASA XPS software, and the binding energies were calibrated using the carbon C 1s peak at 284.6 eV. The XPS survey was used to determine the elemental analysis of the abietates complexes. For Co-abietate the presence of C, O, Na, S, and Co was identified, and for Cu-abietate the presence of C, O, Na, and Cu, as shown in Figure 5.

The relative atomic composition for Co-abietate resulted in carbon (76.0%), oxygen (17.0%), cobalt (5.8%), sodium (0.8%), and sulfur (0.4%), while for Cu-abietate was evaluated as carbon (77.4 %), oxygen (14.5%), copper (3.0%), sodium (4.1%) and sulfur (1.0%). The presence of sodium and sulfur traces in both samples was attributed to the residues from the first and second stages of the synthesis, respectively.

The spectrum of O 1s for the Co-abietate can be reproduced using three components (Gaussians-Lorentzian) centered at 531.2, 532.3, and 533.4 eV corresponding to the Co−O bond, the carbonyl group (C−O), and carboxylate group (O−C=O), respectively. The O 1s spectrum for Cu-abietate is reproduced using five components centered at 531.4; 533.5; 535.4; 538.0; 532.5 eV, associated with Cu−O, the (C−O) bonds, and (O−C=O) bonds, Na KLL Auger, and the sulfur, respectively. The presence of sodium and sulfur atoms only influenced the Cu-abietate sample fitting because it showed a higher percentage of these elements [40,41,42,43].

The C 1s spectrum of Co-abietate shows four components associated with the characteristic peaks of carbon atoms in sp^3^ bonding and carbon atoms bound to hydrogen atoms (C−C/C−H), carbon doubly bound to other carbon (C=C), carbon doubly bound to oxygen (C=O), and carboxylate group (O−C=O), at 284.3, 285.4, 288.0 eV, respectively. In Cu-abietate, four components at 284.3; 285.8; 287.6; 289.9 eV, assigned to the same characteristic peaks attributed in Co-abietate C1s spectrum: C−H/C−C, C=C, C−O/C=O, and O-C=O bonds, respectively [43,44]. All the components denoted in C 1s and O 1s spectra confirm the carboxylate groups presence in the abietates complexes structure, as evidenced in infrared data (FTIR).

### 3.5. Morphological (SEM) Characteristics

**Figure 6:**
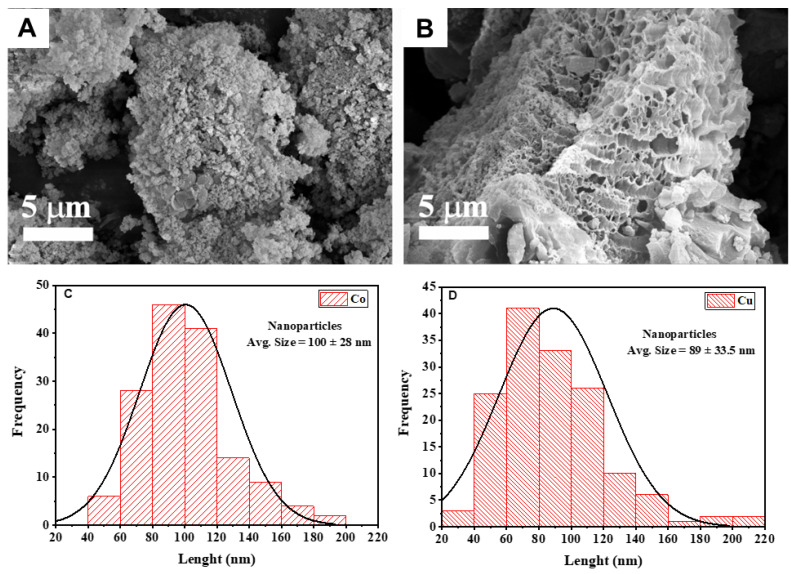
SEM images and average particle sizes of the samples: (**A**,**C**) Co-abietate; (**B**,**D**) Cu-abietate.

The morphology of the abietates complexes was analyzed by scanning electron microscopy (SEM). Figure 6 shows the SEM image of the Co-abietate and Cu-abietate samples. It is observed that the morphology is dependent on the metal core. The Co-abietate morphology is composed of irregular clusters of particles, such as lumps with an ellipsoid to spherical form with an average size of 100 nm (Figure 6C). The surface is homogeneous and rough. There are small vacancies between the particles that tend to prevent the proliferation of bacteria on the surface, as suggested in previous reports [45]. On the other hand, the Cu-abietate morphology shows large rod−shaped pores with slightly non−uniformity, and the surface is rougher than in Co-abietate images. Cu-abietate also showed a smaller particle size than Co-abietate, as shown in Figure 6D. These results indicate that the metal influences the morphology of the complexes.

### 3.6. Colorimetric Analysis

**Figure 7:**
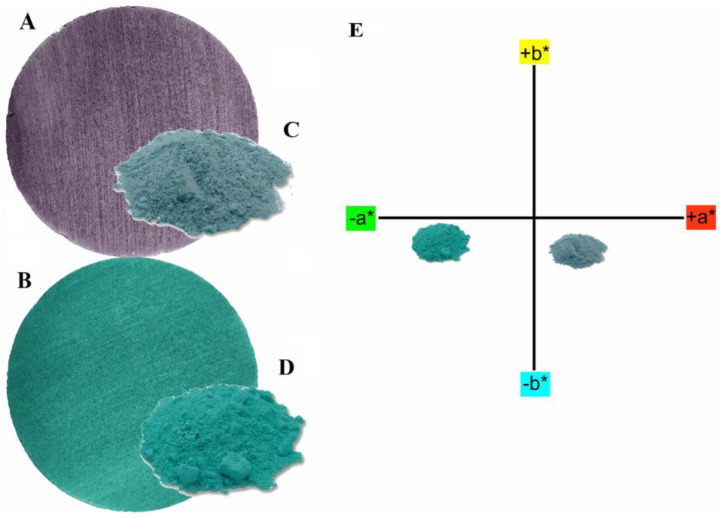
(**A**) specimen painted with pigment Co-abietate dispersed in acrylic paint; (**B**) specimen painted with pigment Cu-abietate dispersed in acrylic paint; (**C**) Co-abietate powder; (**D**) Cu-abietate powder; (**E**) colorimetric parameters a* and b* of the pigments in powder according to CIE L*a*b*.

Figure 7 shows the images of specimens painted with the pigments dispersed in paint (Figure 7A,B), the pigments in powder form (Figure 7C,D), and the colorimetric coordinates of the complexes according to the norm of the International Commission d’Eclairage (CIE) of 1976, which relates the hue and saturation of the materials (Figure 7E). The results obtained through colorimetric coordinates were L* = 73.5; a* = +5.83; b* = −5.31 for Co-abietate, and L* = 76.8; a* = −21.8; b* = −1.93, for Cu-abietate. Co-abietate showed a negative b* value tending to blue, and when combined, the a* coordinate explained the purple color of the powder material. Cu-abietate showed a negative a* value, tending to a green color. These results accord for green materials as expected. Different colors can be observed between the samples according to the chromaticity values, indicating that the metallic core is the main responsible for the color of the complexes. Therefore, different colors can be obtained for their application as synthetic pigments.

### 3.7. TG-DTG

**Figure 8:**
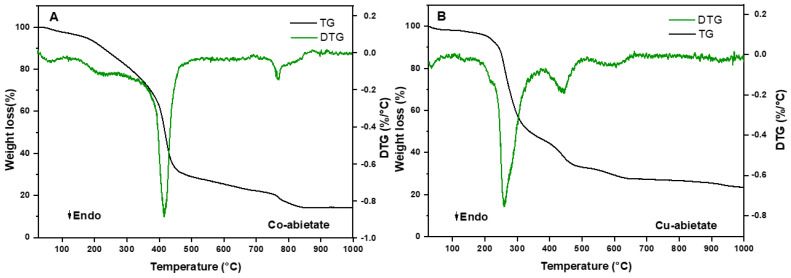
Thermal Decomposition of (**A**) Co-abietate, and (**B**) Cu-abietate samples by TG/DTG.

Thermogravimetric analysis was undertaken to evaluate the thermal stability of the pigments in the temperature range of 25 to 1000 °C. Figure 8 shows the thermogravimetric (TG/DTG) curves for the Co-abietate and Cu-abietate complexes. The thermal decomposition processes were different for each compound. 

The thermal analysis of the Co-abietate, presented in Figure 8A, shows four stages of mass loss. The first stage occurs at 80–124 °C with a mass loss of 3.1% (*m*/*m*) attributed to the loss of water molecules. The second step was observed at 190–280 °C with 14% (*m*/*m*) of organic ligand loss. The third and foremost stage is observed in an interval of 330–490 °C with 45% (*m*/*m*) of mass loss also from the organic ligand loss, and the last step at 710–820 °C occurs with 9% (*m*/*m*) of weight loss due to the decomposition of the COO^−^ a group from the abietate ligands. The mass loss and derivate curves for Cu-abietate (Figure 8B) show that, when compared to the Co-abietate, Cu-abietate has more events, with a total of five stages: the dehydration event occurs from room temperature up to 103 °C, indicating the loss of 1.2% (*m*/*m*), the second is found at 182–363 °C with 49.1% (*m*/*m*) due to ligand decomposition, the third at 182 °C with 1.6% (*m*/*m*) loss related to the abietate decomposition, the fourth occurs at between 363–503 °C with 14.4 % (*m*/*m*) of variation to the ligand decomposition, and the last stage involves 5.6% of mass loss between 503–670 °C, corresponding to the decomposition of the COO^−^ group from the abietate ligands. 

Zhou et al. [46] reported on the decomposition of pine resin + metal compounds showing two prominent stages within the 30–800 °C. In the first, the weight loss below 300 °C was associate with the degradation of the resin. During the second stage, the weight loss was related to the fracture of COO-Metal-OOC bonds. This second step, described by these authors, shows the metals’ influence on the degradation of the complexes from pine resin. The differences in the stages were associated with the different coordination modes, as shown by mass spectrometry and FTIR analysis. According to these authors, the results discussed here suggest that the differences in thermogravimetric (TG/DTG) curves between the complex samples are related to the complexation of the metal in the coordination compounds and the different labilities between the metals. Furthermore, the thermal analysis showed differences between the complexes, even with the same ligand for all the samples. These differences may be due to structural differences between the Co and Cu complexes. 

### 3.8. Antibacterial Activity

Co-abietate and Cu-abietate were tested against the Gram−positive bacterium *Staphylococcus aureus* and the Gram−negative bacterium *Escherichia coli*. Both complexes in DMSO exhibited positive results for *S. aureus* and *E. coli*, with MIC (minimum inhibitory concentration) values of 4.50 µg·mL^−1^, and the concentration range tested was 3.20 to 24.5 µg·mL^−1^. These results are similar to those found in the previous works, as shown in Table 2, and better than those found in Solanki et al. [47], which describes the carboxylates and pyrazole containing mixed ligand copper(II) and cobalt(II) complexes synthesis, which had MIC activity of 200 µg·mL^−1^ against *S. aureus*, and *E.coli*. Reports on the antimicrobial activities of *Pinus* resin [48,49], demonstrated that the *Pinus* resin is insoluble in water, and the pigments (Cu-abietate and Co-abietate) show hydrophobic properties. Hydrophobic coatings can inhibit bacterial growth because they have strong adhesion resistance, which prevents direct contact with the bacteria on the surface [50]. Another explanation for the observed antibacterial properties is the presence of metals (Co and Cu) in synthesizing the pigments that can form reactive oxygen species that inhibit bacterial growth [51]. However, for carboxylates ligands, the antimicrobial effect is likely due to the lipophilic character favoring the interaction with the bacterial cell wall, breaching it, and causing the bacteria’s death [52,53]. An advantage of using metal compounds is that their lifespan is longer than chemical disinfectants because they are not consumed in the inhibition process.

### 3.9. Antiviral Activity

To verify the inhibitory effect of Cu-abietate and Co-abietate against the SARS-CoV-2 virus, the specimens were evaluated using an adaptation of the ISO 21702:2019 guideline for the exposure phase. The remaining intact virus was quantified using an RT-qPCR protocol. The presence of SARS-CoV-2 RNA in environmental samples (Table 3) is used to indicate that virus (viable or nonviable) was present on that surface at some point previously, indicating a viable virus [58]. In the specimens containing Co-abietate and Cu-abietate, the percentage of viral load reduction after 24 h was 99.996% for both samples. These show that pigments at 10% (*W*/*W*) concentrations in commercial paint present an optimal virucidal activity (SARS-CoV-2). Exposure to the acrylic coating (paint matrix) alone resulted in a 1.5 log viral load reduction. Still, the coatings containing Co-abietate and Cu-abietate resulted in a 2.5 log further reduction, proving the efficiency of Co-abietate and Cu-abietate as antiviral pigments. 

The metal complexes have been reported as potential inhibitors of the spike protein of SARS-CoV-2, which in addition to playing a role in the host cell entry, might function as a potential modulator of host immunity to delay or attenuate the immune response against the viruses [58]. This virus belongs to a large family of enveloped viruses with +ssRNA and crown−like spikes on their spherical surfaces [3]. The damage to the protein and envelope of the SARS-CoV-2’s spike destroys the external structure of the virus and thereby inhibits the mechanism by which it infects [20]. Many studies have related that transition metals, such as cobalt and copper, combined with natural diterpenes, such as abietic and dehydroabietic acids (DHAA) from *Pinus* resin, can form efficient complexes to kill viruses. Natural resin is a traditional product of Chinese medicine; its derivatives have a wide range of biological activities [59].

## 4. Discussion on Advantages and Constraints of the Novel Synthesis Process

The use of renewable raw materials to obtain the binder of the complexes contributes to the reduction in production costs and the use of an environmentally friendly product. Furthermore, the proposed synthesis route proved effective at mild temperatures and without organic solvents, which are the main advantages of the proposed green synthesis process. In contrast, the presence of complexes with three and four ligands was identified as shown in mass spectrometry (MS), which could be a possible limitation of the reproducibility of the process since it is not possible to fine-control the formation of a single structure—besides the presence of more than one oxidation state of the metals, indicated by the NEXAFS technique. However, when applied in the coating, the complexes showed good opacity and coating, and the curing process of the coating was not altered. Thus, the complexes have a high potential for application as pigments for architectural paints. In addition, the antibacterial and antiviral properties of the complexes were satisfactory, not requiring antimicrobial additives, leading to a reduction in the cost of the final product.

## 5. Conclusions

We described a novel synthesis route that is low−cost, more straightforward, and environmentally friendly compared to reported ones to obtain similar pigments. The cobalt and copper ions strongly interacted with the ligand, forming stables compounds with a +2 oxidation state in both complexes. It was found that these complexes interact with the carboxylate group in the ligand. These structures were confirmed by elemental analysis, XPS, FTIR spectroscopy, and mass spectrometry. The colorimetric analysis indicated that parameters a* and b* combine purple and green−blue colors for Co-abietate and Cu-abietate, respectively. Both complexes show good thermal stability. The antibacterial test for both complexes showed satisfactory minimum inhibitory concentration (4.50 µg·mL^−1^) against *S. aureus* and *E. coli*. In addition, the samples showed promising results against SARS-CoV-2. Therefore, the synthesized pigments are promising materials to reduce infection proliferation from contact with contaminated surfaces, thus limiting the SARS-CoV-2 spread. Further studies on the toxicity of the pigments will be performed.

## Figures and Tables

**Table 1:** υ_as_ and υ_s_ bands, Δυ, and coordinating mode obtained from the FTIR Spectra.

Sample	υ_as_ (cm^−1^)	υ_s_ (cm^−1^)	Δυ (cm^−1^) *	Coordinating Mode
Na-abietate	1544	1397	147	-
Co-abietate	1570	1411	159	Bidentate
Cu-abietate	1597	1402	195	Unidentate

* Δυ (cm^−1^) = υ_as_(cm^−1^) − υ_s_(cm^−1^).

**Table 2:** MICs from different sources.

Research	Strain	Compound	MIC
[54]	*S. aureus*	[Cu(C_2_H_5_CN)_4_] [B(C_6_F_5_)_4_]	32 (μg/mL)
[55]	*S. aureus* and *E. coli*	Co (II)–PhAlaSal	1.82 (mg/mL)
[56]	*S. aureus* and *E. coli*	[C_22_H_23_CuN_5_O_6_S_2_]2H_2_O	3.9 and 6.63 (μg/mL)
[57]	*E. coli*	[Cu(L)(Cl)(H_2_O)_2_] and [Co(L)(Cl)(H_2_O)_2_]	12.50 (μg/mL)
[47]	*S. aureus* and *E. coli*	Cu(II) and Co(II) complexes	200 (mg/mL)
This work	*S. aureus* and *E. coli*	Co and Cu-abietate	4.50 (μg/mL)

**Table 3:** Decrease in viral load of plates coated with a paint containing Co-abietate and Cu-abietate determined in antiviral testing using an adaptation of the ISO 21702:2019 guideline.

Sample	Decrease (Log)
Control (Copper)	2.90 ^b^
Acrylic paint	1.50 ^c^
Co-abietate	>4 ^a^
Cu-abietate	>4 ^a^

Different lowercase letters in the same row indicate significant differences at *p* ≤ 0.05 by Tukey’s test.

## Data Availability

The data presented in this study are available on request from the corresponding author.

## References

[1] Farhadian S., Heidari-Soureshjani E., Hashemi-Shahraki F., Hasanpour-Dehkordi A., Uversky V.N., Shirani M., Shareghi B., Sadeghi M., Pirali E., Hadi-Alijanvand S. (2022). Identification of SARS-CoV-2 Surface Therapeutic Targets and Drugs Using Molecular Modeling Methods for Inhibition of the Virus Entry. J. Mol. Struct..

[2] Ou X., Liu Y., Lei X., Li P., Mi D., Ren L., Guo L., Guo R., Chen T., Hu J. (2020). Characterization of Spike Glycoprotein of SARS-CoV-2 on Virus Entry and Its Immune Cross-Reactivity with SARS-CoV. Nat. Commun..

[3] Mizielińska M., Nawrotek P., Stachurska X., Ordon M., Bartkowiak A. (2021). Packaging Covered with Antiviral and Antibacterial Coatings Based on Zno Nanoparticles Supplemented with Geraniol and Carvacrol. Int. J. Mol. Sci..

[4] Viana Martins C.P., Xavier C.S.F., Cobrado L. (2022). Disinfection Methods against SARS-CoV-2: A Systematic Review. J. Hosp. Infect..

[5] Ayub M., Othman M.H.D., Khan I.U., Yusop M.Z.M., Kurniawan T.A. (2021). Graphene-Based Nanomaterials as Antimicrobial Surface Coatings: A Parallel Approach to Restrain the Expansion of COVID-19. Surf. Interfaces.

[6] Lin N., Verma D., Saini N., Arbi R., Munir M., Jovic M., Turak A. (2021). Antiviral Nanoparticles for Sanitizing Surfaces: A Roadmap to Self-Sterilizing against COVID-19. Nano Today.

[7] Percivalle E., Clerici M., Cassaniti I., Vecchio Nepita E., Marchese P., Olivati D., Catelli C., Berri A., Baldanti F., Marone P. (2021). SARS-CoV-2 Viability on Different Surfaces after Gaseous Ozone Treatment: A Preliminary Evaluation. J. Hosp. Infect..

[8] Imani S.M., Ladouceur L., Marshall T., Maclachlan R., Soleymani L., Didar T.F. (2020). Antimicrobial Nanomaterials and Coatings: Current Mechanisms and Future Perspectives to Control the Spread of Viruses Including SARS-CoV-2. ACS Nano.

[9] Minoshima M., Lu Y., Kimura T., Nakano R., Ishiguro H., Kubota Y., Hashimoto K., Sunada K. (2016). Comparison of the Antiviral Effects of Solid-State Copper and Silver Compounds. J. Hazard. Mater..

[10] Besold A.N., Culbertson E.M., Culotta V.C. (2016). The Yin and Yang of Copper during Infection. J. Biol. Inorg. Chem..

[11] Shionoiri N., Sato T., Fujimori Y., Nakayama T., Nemoto M., Matsunaga T., Tanaka T. (2012). Investigation of the Antiviral Properties of Copper Iodide Nanoparticles against Feline Calicivirus. J. Biosci. Bioeng..

[12] Delehanty J.B., Bongard J.E., Thach D.C., Knight D.A., Hickey T.E., Chang E.L. (2008). Antiviral Properties of Cobalt(III)-Complexes. Bioorg. Med. Chem..

[13] Rani I., Goyal A., Bhatnagar M., Manhas S., Goel P., Pal A., Prasad R. (2021). Potential Molecular Mechanisms of Zinc- and Copper-Mediated Antiviral Activity on COVID-19. Nutr. Res..

[14] Panda S., Deshmukh K., Mustansar Hussain C., Khadheer Pasha S.K. (2022). 2D MXenes for Combatting COVID-19 Pandemic: A Perspective on Latest Developments and Innovations. FlatChem.

[15] Rastogi A., Singh A., Naik K., Mishra A., Chaudhary S., Manohar R., Singh Parmar A. (2022). A Systemic Review on Liquid Crystals, Nanoformulations and Its Application for Detection and Treatment of SARS-CoV-2 (COVID-19). J. Mol. Liq..

[16] Ghasemi L., Hasanzadeh Esfahani M., Abbasi A., Behzad M. (2022). Synthesis and Crystal Structures of New Mixed-Ligand Schiff Base Complexes Containing N-Donor Heterocyclic Co-Ligands: Molecular Docking and Pharmacophore Modeling Studies on the Main Proteases of SARS-CoV-2 Virus (COVID-19 Disease). Polyhedron.

[17] Tretyakova E.V., Smirnova I.E., Salimova E.V., Odinokov V.N. (2015). Synthesis and Antiviral Activity of Maleopimaric and Quinopimaric Acids’ Derivatives. Bioorg. Med. Chem..

[18] De Oliveira Junkes C.F., Duz J.V.V., Kerber M.R., Wieczorek J., Galvan J.L., Fett J.P., Fett-Neto A.G. (2019). Resinosis of Young Slash Pine (*Pinus elliottii* Engelm.) as a Tool for Resin Stimulant Paste Development and High Yield Individual Selection. Ind. Crops Prod..

[19] Ryu Y.B., Jeong H.J., Kim J.H., Kim Y.M., Park J.Y., Kim D., Naguyen T.T.H., Park S.J., Chang J.S., Park K.H. (2010). Biflavonoids from Torreya Nucifera Displaying SARS-CoV 3CLpro Inhibition. Bioorg. Med. Chem..

[20] Geromichalou E.G., Trafalis D.T., Dalezis P., Malis G., Psomas G., Geromichalos G.D. (2022). In Silico Study of Potential Antiviral Activity of Copper(II) Complexes with Non–Steroidal Anti–Inflammatory Drugs on Various SARS–CoV–2 Target Proteins. J. Inorg. Biochem..

[21] Karges J., Cohen S.M. (2021). Metal Complexes as Antiviral Agents for SARS-CoV-2. ChemBioChem.

[22] De Souza Correa J., dos Santos R.R., Anaissi F.J. (2018). Purification and Characterization of Colophony Extracted of *Pinus elliottii* (Engelm, Var. Elliottii). Orbital.

[23] Correa J.S., Primo J.O., Bittencourt C., Horsth D.F.L., Radovanovic E., Silveira-Jr A.T., Toma H.E., Zanette C.M., Anaissi F.J. (2022). Experimental Data for Green Synthesis of Zn-Abietate Complex from Natural Resin. Data Br..

[24] Correa J.S., Primo J.O., Bittencourt C., Horsth D.F.L., Radovanovic E., Silveira-Jr A.T., Toma H.E., Zanette C.M., Anaissi F.J. (2022). Ecofriendly Synthesis of Zn-Abietate Complex Derived from *Pinus elliottii* Resin and Its Application as an Antibacterial Pigment against *S. aureus* and *E. coli*. Dye. Pigment..

[25] Guttmann P., Werner S., Siewert F., Sokolov A., Schmidt J.-S., Mast M., Brzhezinskaya M., Jung C., Follath R., Schneider G. (2018). The New HZB X-Ray Microscopy Beamline U41-PGM1-XM at BESSY II. Microsc. Microanal..

[26] Werner S., Guttmann P., Siewert F., Sokolov A., Mast M., Huang Q., Feng Y., Li T., Senf F., Follath R. (2023). Spectromicroscopy of Nanoscale Materials in the Tender X-Ray Regime Enabled by a High Efficient Multilayer-Based Grating Monochromator. Small Methods.

[27] Cheung V. (2016). Uniform Color Spaces. Handbook of Visual Display Technology.

[28] Clinical and Laboratory Standards Institute (2015). M02-A12: Performance Standards for Antimicrobial Disk Susceptibility Tests; Approved Standard—Twelfth Edition. Clin. Lab. Stand. Inst..

[29] Papageorgiou S.K., Kouvelos E.P., Favvas E.P., Sapalidis A.A., Romanos G.E., Katsaros F.K. (2010). Metal-Carboxylate Interactions in Metal-Alginate Complexes Studied with FTIR Spectroscopy. Carbohydr. Res..

[30] Deacon G.B., Phillips R.J. (2005). Relationships between the Carbon-Oxygen Stretching Frequencies of Carboxylate Complexes and the Type of Carboxylate Coordination. Coord. Chem. Rev..

[31] Palacios E.G., Juárez-López G., Monhemius A.J. (2004). Infrared Spectroscopy of Metal Carboxylates: II. Analysis of Fe(III), Ni and Zn Carboxylate Solutions. Hydrometallurgy.

[32] Neuville D.R., Cormier L., Flank A.-M., Prado R.J., Lagarde P. (2004). Na K-Edge XANES Spectra of Minerals and Glasses. Eur. J. Mineral..

[33] Ragoen C., Cormier L., Bidegaray A.I., Vives S., Henneman F., Trcera N., Godet S. (2018). A XANES Investigation of the Network-Modifier Cations Environment before and after the Na^+^/K^+^ Ion-Exchange in Silicate Glasses. J. Non-Cryst. Solids.

[34] Singh J.P., Gautam S., Lim W.C., Asokan K., Singh B.B., Raju M., Chaudhary S., Kabiraj D., Kanjilal D., Lee J.M. (2017). Electronic Structure of Magnetic Fe/MgO/Fe/Co Multilayer Structure by NEXAFS Spectroscopy. Vacuum.

[35] Zhu D., Cao Q., Qiao R., Zhu S., Yang W., Xia W., Tian Y., Liu G., Yan S. (2016). Oxygen Vacancies Controlled Multiple Magnetic Phases in Epitaxial Single Crystal Co_0.5_(Mg_0.55_Zn_0.45_)_0.5_O_1-v_ Thin Films. Sci. Rep..

[36] Wu Y., Chen Z., Cheong W.C., Zhang C., Zheng L., Yan W., Yu R., Chen C., Li Y. (2019). Nitrogen-Coordinated Cobalt Nanocrystals for Oxidative Dehydrogenation and Hydrogenation of N-Heterocycles. Chem. Sci..

[37] Istomin S.Y., Tyablikov O.A., Kazakov S.M., Antipov E.V., Kurbakov A.I., Tsirlin A.A., Hollmann N., Chin Y.Y., Lin H.J., Chen C.T. (2015). An Unusual High-Spin Ground State of Co^3+^ in Octahedral Coordination in Brownmillerite-Type Cobalt Oxide. Dalt. Trans..

[38] Gurevich A.B., Bent B.E., Teplyakov A.V., Chen J.G. (1999). NEXAFS Investigation of the Formation and Decomposition of CuO and Cu_2_O Thin Films on Cu(100). Surf. Sci..

[39] Sivkov D.V., Petrova O.V., Nekipelov S.V., Vinogradov A.S., Skandakov R.N., Isaenko S.I., Ob’edkov A.M., Kaverin B.S., Vilkov I.V., Korolev R.I. (2021). The Identification of Cu–o–c Bond in Cu/Mwcnts Hybrid Nanocomposite by Xps and Nexafs Spectroscopy. Nanomaterials.

[40] Wu Z.W., Tyan S.L., Chen H.H., Huang J.C.A., Huang Y.C., Lee C.R., Mo T.S. (2017). Temperature-Dependent Photoluminescence and XPS Study of ZnO Nanowires Grown on Flexible Zn Foil via Thermal Oxidation. Superlattices Microstruct..

[41] Biesinger M.C., Lau L.W.M., Gerson A.R., Smart R.S.C. (2010). Resolving Surface Chemical States in XPS Analysis of First Row Transition Metals, Oxides and Hydroxides: Sc, Ti, V, Cu and Zn. Appl. Surf. Sci..

[42] Bedar A., Tewari P.K., Bindal R.C., Kar S. (2020). Enhancing γ-Radiation Resistant Property of Polysulfone Membranes with Carboxylated Nanodiamond: Impact and Effect of Surface Tunability. Appl. Surf. Sci..

[43] Carvalho M.F.N.N., Botelho do Rego A.M., Galvão A.M., Herrmann R., Marques F. (2018). Search for Cytotoxic Compounds against Ovarian Cancer Cells: Synthesis, Characterization and Assessment of the Activity of New Camphor Carboxylate and Camphor Carboxamide Silver Complexes. J. Inorg. Biochem..

[44] Lopez T., Cuevas J.L., Ilharco L., Ramírez P., Rodríguez-Reinoso F., Rodríguez-Castellón E. (2018). XPS Characterization and *E. coli* DNA Degradation Using Functionalized Cu/TiO_2_ Nanobiocatalysts. Mol. Catal..

[45] Dias C.O., dos Santos Opuski de Almeida J., Pinto S.S., de Oliveira Santana F.C., Verruck S., Müller C.M.O., Prudêncio E.S., de Mello Castanho Amboni R.D. (2018). Development and Physico-Chemical Characterization of Microencapsulated Bifidobacteria in Passion Fruit Juice: A Functional Non-Dairy Product for Probiotic Delivery. Food Biosci..

[46] Zhou W., Wang Y., Ni C., Yu L. (2021). Preparation and Evaluation of Natural Rosin-Based Zinc Resins for Marine Antifouling. Prog. Org. Coatings.

[47] Solanki A., Sadhu M.H., Patel S., Devkar R., Kumar S.B. (2015). Ternary Complexes of Copper(II) and Cobalt(II) Carboxylate with Pyrazole Based Ligand: Syntheses, Characterization, Structures, and Bioactivities. Polyhedron.

[48] Li Z., Yang X., Liu H., Yang X., Shan Y., Xu X., Shang S., Song Z. (2019). Dual-Functional Antimicrobial Coating Based on a Quaternary Ammonium Salt from Rosin Acid with in Vitro and in Vivo Antimicrobial and Antifouling Properties. Chem. Eng. J..

[49] Kong Q., Li Z., Ding F., Ren X. (2021). Hydrophobic N-Halamine Based POSS Block Copolymer Porous Films with Antibacterial and Resistance of Bacterial Adsorption Performances. Chem. Eng. J..

[50] Motshekga S.C., Sinha Ray S., Maity A. (2018). Synthesis and Characterization of Alginate Beads Encapsulated Zinc Oxide Nanoparticles for Bacteria Disinfection in Water. J. Colloid Interface Sci..

[51] Hikku G.S., Jeyasubramanian K., Vignesh Kumar S. (2017). Nanoporous MgO as Self-Cleaning and Anti-Bacterial Pigment for Alkyd Based Coating. J. Ind. Eng. Chem..

[52] Turlybekuly A., Pogrebnjak A.D., Sukhodub L.F., Sukhodub L.B., Kistaubayeva A.S., Savitskaya I.S., Shokatayeva D.H., Bondar O.V., Shaimardanov Z.K., Plotnikov S.V. (2019). Synthesis, Characterization, in Vitro Biocompatibility and Antibacterial Properties Study of Nanocomposite Materials Based on Hydroxyapatite-Biphasic ZnO Micro- and Nanoparticles Embedded in Alginate Matrix. Mater. Sci. Eng. C.

[53] Zelenák V., Györyová K., Mlynarcík D. (2001). Antibacterial and Antifungal Activity of Zinc(II) Carboxylates with/without N-Donor Organic Ligands. Met. Based. Drugs.

[54] Hijazi A.K., Taha Z.A., Abuhamad N.J., Al-Momani W.M. (2021). Synthesis, and Characterization of Some Cu(I) Complexes Having Propionitrile and Pyridine Moieties: An Investigation on Their Antibacterial Properties. J. Saudi Chem. Soc..

[55] Woźniczka M., Świątek M., Sutradhar M., Gądek-Sobczyńska J., Chmiela M., Gonciarz W., Pasternak B., Pająk M. (2023). Equilibria of Complexes in the Aqueous Cobalt(II)–N-(2-Hydroxybenzyl)Phenylalanine System and Their Biological Activity Compared to Analogous Schiff Base Structures. Comput. Struct. Biotechnol. J..

[56] Ragab A., Ammar Y.A., Ezzat A., Mahmoud A.M., Mohamed M.B.I., El-Tabl A.S., Farag R.S. (2022). Synthesis, Characterization, Thermal Properties, Antimicrobial Evaluation, ADMET Study, and Molecular Docking Simulation of New Mono Cu (II) and Zn (II) Complexes with 2-Oxoindole Derivatives. Comput. Biol. Med..

[57] Yernale N.G., Matada B.S., Vibhutimath G.B., Biradar V.D., Karekal M.R., Udayagiri M.D., Hire Mathada M.B. (2022). Indole Core-Based Copper(II), Cobalt(II), Nickel(II) and Zinc(II) Complexes: Synthesis, Spectral and Biological Study. J. Mol. Struct..

[58] Paton S., Spencera A., Garratta I., Thompsona K.-A., Dinesha I., Aranega-Boua P., Stevensona D., Clarka S., Dunningb J., Bennetta A. (2021). Persistence of Severe Acute Respiratory Syndrome Coronavirus 2 (SARS-CoV-2) Virus and Viral RNA in Relation to Surface Type and Contamination Concentration. Appl. Environ. Microbiol..

[59] Fei B.L., Tu S., Wei Z., Wang P., Qiao C., Chen Z.F. (2019). Optically Pure Chiral Copper(II) Complexes of Rosin Derivative as Attractive Anticancer Agents with Potential Anti-Metastatic and Anti-Angiogenic Activities. Eur. J. Med. Chem..

